# Constitutive versus Responsive Gene Expression Strategies for Growth in Changing Environments

**DOI:** 10.1371/journal.pone.0027033

**Published:** 2011-11-30

**Authors:** Nico Geisel

**Affiliations:** Departament de Fisica Fonamental, Facultat de Fisica, Universitat de Barcelona, Barcelona, Spain; Mount Sinai School of Medicine, United States of America

## Abstract

Microbes respond to changing environments by adjusting gene expression levels to the demand for the corresponding proteins. Adjusting protein levels is slow, consequently cells may reach the optimal protein level only by a time when the demand changed again. It is therefore not a priori clear whether expression “on demand” is always the optimal strategy. Indeed, many genes are constitutively expressed at intermediate levels, which represents a permanent cost but provides an immediate benefit when the protein is needed. Which are the conditions that select for a responsive or a constitutive expression strategy, what determines the optimal constitutive expression level in a changing environment, and how is the fitness of the two strategies affected by gene expression noise? Based on an established model of the *lac-* and *gal-*operon expression dynamics, we study the fitness of a constitutive and a responsive expression strategy in time-varying environments. We find that the optimal constitutive expression level differs from the average demand for the gene product and from the average optimal expression level; depending on the shape of the growth rate function, the optimal expression level either provides intermediate fitness in all environments, or maximizes fitness in only one of them. We find that constitutive expression can provide higher fitness than responsive expression even when regulatory machinery comes at no cost, and we determine the minimal response rate necessary for “expression on demand” to confer a benefit. Environmental and inter-cellular noise favor the responsive strategy while reducing fitness of the constitutive one. Our results show the interplay between the demand-frequency for a gene product, the genetic response rate, and the fitness, and address important questions on the evolution of gene regulation. Some of our predictions agree with recent yeast high throughput data, for others we propose the experiments that are needed to verify them.

## Introduction

In natural environments cells are frequently facing variable conditions, to which they must adapt in order to maximize growth and survival. Common environmental parameters subject to fluctuations are the kinds of nutrient that are available, the temperature, the salt content of the surroundings, and the concentration of toxins and antibiotics.

Understanding microbial behaviors in changing environments provides insights into the evolution in natural habitats where the physiologic demands are constantly changing [1]–[3]. Manipulation of these strategies can be relevant in industrial processing, e.g. fermentation [4], antibiotic therapies [5] and biotechnological process optimization.

Prokaryotes and eukaryotes cope with environmental changes by switching between different gene-expression states (phenotypes) [2], [3], [6]–[9], typically accompanied by metabolic and morphologic changes [7], [10], [11]. A particular phenotype provides a growth or survival advantage in one environmental condition, but is maladapted in other environments. The most prominent examples are the vegetative and persistent states of bacterial populations [2], [3], [12]–[14]. In the vegetative state cells can rapidly proliferate but are highly vulnerable to antibiotic stress. In the persistent state, on the other hand, they can survive antibiotic exposure but cannot divide. Similar situations arise for pili-expression and at the level of metabolic systems: production of *lacZ* is energetically costly and reduces *E. coli's* growth rate in the absence of lactose [15]–[18]. When lactose is the only energy source, in turn, production of *lacZ* enhances growth [16], [19], [20].

How microbial populations maximize their time-averaged growth rate in a changing environment has been investigated experimentally and theoretically along two major lines [2], [6], [21]–[26]. In the responsive switching strategy all cells switch into the adapted state upon an environmental change. With stochastic switching a population follows a bet-hedging strategy because cells also transit randomly into maladapted states. Thereby the population maintains a small maladapted subpopulation which may be well-adapted and ready for growth after a future environmental change. Previous studies were based on the assumption that cellular phenotype transitions occur stochastically at a given rate (also in the responsive case). Therefore switching is modeled as an instantaneous event which, however, occurs after a random delay [2], [3], [6], [21], [23]–[25]. Accordingly, cells exist only in two states (fit, unfit) but never in the transient states of adaptation, between the unfit and the fit phenotype. An implicit assumption is that the time intervals between switching events are very large, i.e., transitions occur only once in many generations [23].

Most phenotypic transitions, however, are responsive and take several hours, in particular if large scale metabolic and morphologic changes are involved [5], [10], [11]. They proceed through a sequence of intermediate states where the fit state is upregulated while the unfit phenotype is downregulated [5], [17], [27]–[29]. When the time scale of phenotypic switching (adaptation) is comparable to the environmental durations the states of intermediate adaptation become relevant for the total fitness and should therefore be taken into account - unlike a two phenotype (fit, unfit) scenario. Under these considerations it appears that a third strategy to cope with environmental fluctuations is a passive “intermediate” one, where cells constitutively express an intermediate phenotype in all environments. Indeed, this strategy appears to be widely used since many procaryotic and eucaryotic genes are constitutively expressed although the demand for expression varies in time. Given that regulated gene expression is adaptive by definition, it is not a priori clear why constitutive expression can provide an advantage. What then determines whether a gene should be under regulated or constitutive expression?

The focus of this article is to understand how environmental factors determine the optimal constitutive expression levels that maximizes net growth in a changing environment, and to understand why and under which conditions constitutive expression confers a growth advantage compared to regulated, responsive expression.

To answer these questions we propose a model that builds on previously established descriptions of the *lac-* and *gal-* operon expression dynamics [17], [22], [30], and compare the time-averaged growth rates of both strategies in a two-state environment, taking account of environmental and inter-cellular noise.

We find that the optimal constitutive expression level depends on how the costs and benefits increase with the expression level: in one case growth is maximized be constitutively expressing the gene at an intermediate level and in the other case the gene is either fully expressed or fully repressed. Surprisingly, the optimum constitutive expression level in a changing environment is always different from the time-averaged demand for the gene product. We find that a responsive strategy can have lower fitness than a constitutive strategy even when the cost for sensing and regulatory machinery is neglected, and we determine the minimal adaptation rate necessary for a response to confer a benefit over constitutive expression. Environmental and inter-cellular noise favor the responsive strategy, whereas they decrease the fitness of the constitutive strategy. Our analysis illustrates the interplay between demand-frequency for a gene product, maladaptation cost, and the time scale of a genetic response, and it raises important questions on the evolution of gene expression strategies.

## Methods

**Figure 1:**
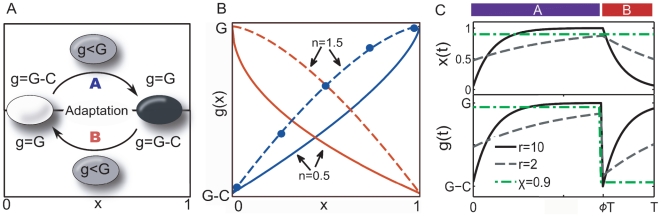
Model for cellular growth and adaptation of the expression state *x(t)* in a two-state environment. (A) In environment 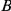 (bottom) the expression state 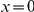 (‘off’) allows for proliferation at the highest rate 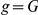. Upon an environmental change 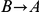 (top) the state 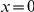 is maladapted and the population grows at a reduced rate 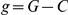, where 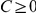 is the cost of maladaptation. In the adaptation phase (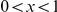, curved arrows) cells suppress the unfit phenotype and continuously upregulate the fit one. This increases their growth rate 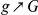 until they are fully adapted to the new environment (for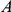: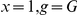). We also consider a constitutive-passive strategy where cells maintain a constant state 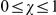 throughout all times in both environments. (B) Growth rate as a function of the expression state 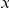 in environment 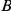 (red) and in environment 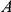 (blue). Dots show the experimentally measured [16] benefit of *E. coli* expressing the *lac-*operon at a fraction 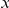 of the optimal level (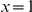) at 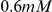 lactose. We generalize this cost function to account for convex (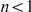, full lines) or concave (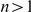 dashed lines) dependence. (C) Adaptation dynamics 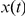 (top) and growth rates 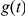 (bottom) in an environmental cycle 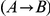. The full black line corresponds to a population which responds ten times faster than the environmental frequency (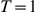) and which therefore tracks the environmental change 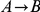 occurring at 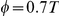, eventually reaching the adapted states. The gray dashed line corresponds to a slowly-adapting population (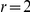) which never reaches the adapted states and instead oscillates around an intermediate expression level. The constitutive-passive population (dashed-green line, 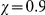) has a high growth rate in environment 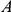 during 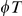, but a small one in 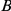 during 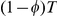.

We propose a model based on the expression dynamics of metabolic operons as described in [16], [17], [22], [31]. We denote the expression state of a cell by 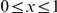, where the fully induced state 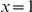 is optimal (maximizing the growth rate) in the environment 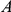 whereas the repressed state 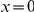 denotes a phenotype that is optimal in environment 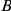 see Figure 1A [16], [22]. Upon an environmental change a population adapts by responsively switching either into the ‘on’ or the ‘off’ state (curved arrows). For many systems these transitions follow an exponential relaxation [17], [22], [30]–[33]. With the adapted states being 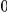 and 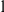 and a relaxation rate 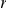 this is modeled by 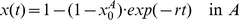
(1)




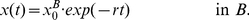
(2)


**Figure 2:**
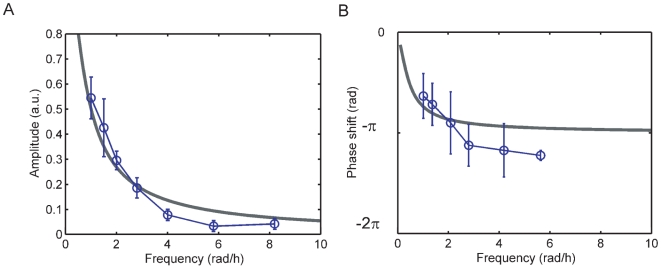
Amplitude-response and phase shift of the model compared to the Yeast YPH499 *gal-*operon. We define the phase shift in our model as twice the time required to reach the half-maximum expression level of 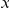 (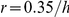). The model according to Eq. 1 and Eq. 2 mimics the galactose utilization network response over a broad frequency range (data points as measured in [34]). The deviation of the experimental phase shift from the predicted phase shift at high frequencies indicates that the response does not exactly follow an exponential relaxation. Indeed, the feedback architecture of the *gal-*network may give rise to short delays which become noticeable at high cycle frequencies (phase shifts 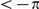), which we do not take into account in our model.

Here 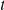 refers to the time since the last environmental change and 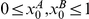 is the expression state with which the population enters into a new environment. This model accurately reproduces the amplitude and phase shift response of the *gal*-operon to external glucose driving (over a galactose background) with different frequencies, as measured in [34] (see Figure 2).

Cells in the optimal state grow at a maximal rate 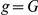, whereas suboptimal states confer inferior growth rates 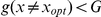 [2, 3 16, 17, 19, 22]. The dots in Figure 1B show the growth benefit of *E. coli* under the assumption that the lac operon is induced at a fraction 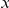 of the optimal induction level in a constant 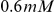 lactose environment (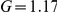) (data points as measured in [16]). As a generalization we assume that the reduction of the growth rate when not in the optimal state is proportional to cost-constants 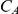 or 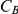 (depending on the environment) and that it depends monotonously on the expression state 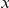 with exponents 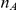 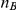 (as recently suggested in [35]): 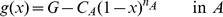
(3)




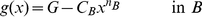
(4)


Here 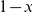, or 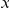 respectively, is the deviation from the optimal phenotype in a given environment. The parameters 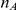 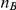 allow for convex or concave dependence of the growth rate on the expression level [16], e.g., for the benefit (cost) of producing a metabolic enzyme in the presence (absence) of its substrate. Figure 1B illustrates these relationships for environment 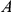 (in blue) and environment 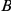 (in red) with 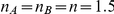 (dashed lines) and 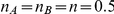 (full lines). In contrast to previous studies [6], [33] we make the important but plausible assumption that the cost for sensing and signaling machinery is negligible. We thus focus only on the dynamical aspects of the response.

A passive population constitutively expresses the same phenotype 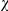 throughout all environments. Equations 3 and 4 then apply with 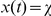.

Figure 1C (top) shows the adaptation dynamics of the phenotype 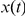 (top panel) and of the growth rate 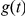 (bottom panel) according to Eq. 1 to Eq. 4. The environment changes from 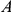 to 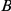 at 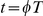 where 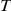 is the total duration of the environmental cycle and 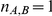.

*E. coli* and other procaryotes are believed to be optimized for fast growth. We therefore take the time-averaged growth rate 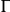 as a measure of fitness in the changing environment [6], [22]–[25]. Without loss of generality we assume that an environmental cycle starts with condition 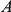 lasting for a time 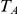, and ends with environment 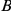 of duration 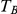 (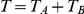). The time-averaged growth rates 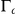 and 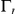 of the constitutive and responsive populations are obtained by integrating Eq. 3 and Eq. 4 over the duration 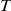 of a full cycle: 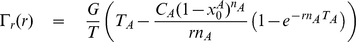


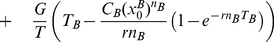
(5)

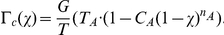


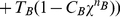
(6)


The second terms in the parentheses of Eq. 5 are the integrated costs during the adaptation phase towards the fit state, and decrease with the response rate 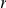.

It is instructive to first consider periodic environmental cycles and we chose the cycle duration as the reference time scale 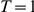, with 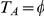 and 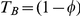. In the periodic case the up-and-downregulation dynamics of 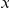
will eventually become periodic with the phenotypic states 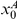 at the end of 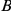 (beginning of 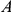), and 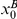 at the end of 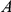 given by 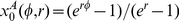
(7)

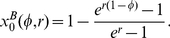
(8)


These correspond to the fixed points when propagating the expression state according to Eq. 1 and Eq. 2 over one cycle. From now on we will assume that maladaptation and growth rate function are symmetric in both environments (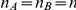 and 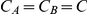) and set the maximal growth rate 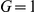.

## Results

### Optimal constitutive expression levels in a time-varying environment

**Figure 3:**
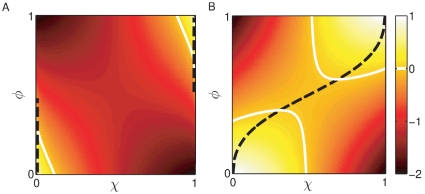
Time-averaged growth rates 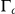 of constitutive populations in periodic environments. The fitness is shown as a function of the constitutive expression level 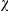 and of the fraction 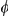 of environment 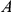 (the environment which requires expression). Regimes of positive net-growth are delineated by the white line (maladaptation cost 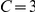). Left and right panels show the time-averaged growth rate for a convex (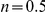) and a concave (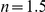) growth rate function. The optimal constitutive expression level is indicated by the dashed line. For a convex or linear dependence (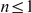) an all-or-nothing strategy with maximal growth in one environment and no growth in the other is optimal. In striking contrast, however, for a concave dependence 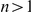 an intermediate strategy with suboptimal growth in both environments is best. In both cases the optimal constitutive level is different from the average optimum, and from the average demand for expression (i.e., the diagonal 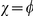). Note that the constitutive strategy can only provide growth when it is close to its optimum *and* when the environment is sufficiently constant (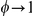 or 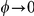). In symmetric environments (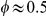) no positive net-growth is possible, hence regulation becomes imperative in this regime.

As a measure of fitness we determine the time-averaged growth rate of the constitutive strategy (see Eq. 6) which is shown in Figure 3 (color coded) in a periodic environment. The constitutive phenotype 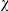 is shown on the x-axis and the fraction 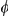 of environment 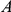 (the demand for expression) is shown on the y-axis. Panel A shows the fitness for 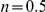 and panel B for 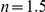. The maladaptation cost is 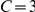, thus in significantly maladapted states the population has a negative growth rate. The white lines delineate the regimes in which the net-growth rate is positive. The dashed curves show the optimal constitutive phenotypes 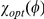 that maximize the time-averaged growth rate.

Interestingly, when the growth rate 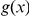 is a linear or convex function (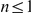), the optimal constitutive strategy is an *all-or-nothing* strategy. In this case net-growth in a changing environment is maximized by maximizing growth in the prevailing environment while growth is minimal in the other, cf. Figure 3A. In contrast, the optimal strategy is an *intermediate* one, with intermediate fitness in both environments (cf. Figure 3B), only when the growth rate is a concave function (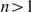, as for the benefit of *lac-*expression). In general, and contrary to what one might have expected, the optimal constitutive phenotype in a time-varying environment does not correspond to the time-averaged demand for this phenotype nor to the average optimum, i.e., a phenotype 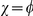 has significantly inferior net fitness compared to 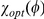.

When none of the environments prevails (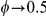), the constitutive strategy cannot provide growth. A passive strategy is therefore not an option at high maladaptation costs 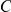 and when both environments are equally frequent, making responsive expression regulation an imperative in this regime. We mention in addition that for 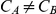 the curve 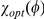 of optimal expression is stretched towards higher (lower) expression, whereas it becomes highly nonlinear and step-like for 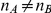.

### Constitutive expression can provide higher fitness than regulated expression

**Figure 4:**
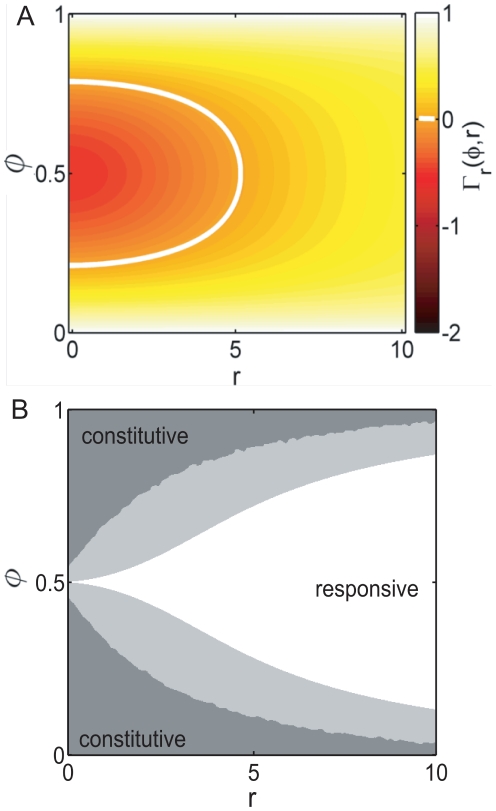
Fitness of a responsive population (A) and strategy phase diagram (B). (A) The responsive population has negative growth 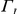 when its response rate 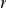 is too small; the growth-threshold is indicated by the white line. The net maladaptation cost is largest at 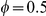 (when both environments have equal durations) because in this regime the population spends most of the time transiting between adapted states rather than being adapted. (B) shows the regimes of optimal strategy (constitutive or responsive) as a function of the demand for expression 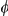 (environment 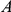) and response rate 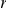.The regime in which a responsive strategy with rate 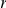 confers higher fitness than a constitutive strategy is indicated in white, and for a stochastic environment in light gray and white. When environments are asymmetric 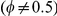 a slow responsive population lags behind the environment and cannot reach an adapted state in any of the two conditions. Therefore it has lower fitness than the constitutive strategy which provides immediate although intermediate growth in both environments. The phase boundaries are independent of the maladaptation cost 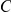.

Similarly as net proliferation requires that the passive population is sufficiently well adapted, the responsive population can only achieve a positive net grow rate at high maladaptation costs if the response rate 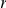 lies above a threshold, cf. the white line in Figure 4A (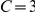). When the environment spends equal amounts of time in 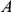 as in 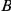 (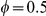) the population spends significant amounts of time transiting between phenotypes rather than in the adapted phenotypes, which reduces the time-averaged growth rate. In particular, when the response rate is too small the population never reaches the adapted state, but instead low-pass filters the environmental change and slowly oscillates around a phenotype 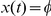, which corresponds to the time-averaged demand, see also Figure 1C (dashed gray line) and [34]. When the environmental durations are asymmetric (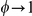 or 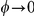) the population remains partially adapted to the predominant environment in the environment of short duration. The population thereby has a lower growth rate in the sporadic environment, but achieves a higher *average* growth rate.

Gene expression levels can be adjusted to their optimum by a few point mutations and within a few hundred generations [16]. We therefore make the plausible assumption that the constitutive population is optimally adapted to an environmental cycle, i.e. 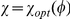. Since the responsive strategy follows environmental changes and approaches the optimum state in a given environment, a response should always confer superior growth than constitutive expression. Figure 4B compares the time-averaged growth rate of the constitutive strategy with a responsive strategy of adaptation rate 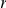 (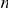 = 1). There exist three regimes indicating whether a constitutive or a responsive strategy confers faster growth. The white area in Figure 4B encloses the regime in which the responsive population has a higher time-averaged growth rate. When the response rate highly exceeds the environmental rate of change the population follows the environment quasi-instantaneously and is quasi-always adapted.

Remarkably, however, as 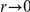, the time-averaged growth rate of the responsive population becomes smaller than the one of the constitutively expressing population (indicated by the gray shaded areas). In particular in asymmetric environments (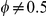) the constitutive population can achieve superior growth even when the response rate is ten times larger than the environmental frequency. Consequently, responding to environmental changes provides a benefit only if the response rate lies above a threshold. Interestingly this suggests that a fast response cannot evolve from constitutive expression via a slow response because fitness along this path would have lower than constitutive fitness.

The slower growth of the responsive population is a consequence of the low-pass filtering which occurs when the adaptation time 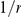 is longer than the duration of the short environment. As explained above, the phenotypic state slowly oscillates around 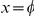 (the average demand) which is suboptimal compared to the constitutive level 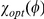. In an asymmetric environment the sporadic condition drives the responsive population away from the state which is adapted in the prevailing condition. The responsive population therefore cannot reach the adapted state in any of the two environments. The constitutive population, on the other hand, benefits from having intermediate growth without a delay in both environments(at 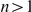), or maximal growth in the prevailing environment (at 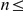).

Importantly, the phase boundaries are independent of the maladaptation cost 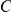. Without going into details, we point out, that when 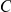 is large there exist two regimes in which the passive population has a positive net-growth rate, whereas the responsive one has a negative net growth rate. When the maladaptation costs are different in the two environments, the phase boundaries become asymmetric and are shifted along 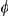 and the maximal growth benefit at a given response rate 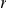 decreases compared to the symmetric case 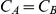, rendering constitutive expression even more favorable. For a convex dependence on the phenotype (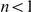) the phase boundary is shifted to larger response rates (because the growth rate 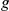 relaxes slower than the expression state 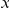), whereas it moves to smaller response rates for a concave dependence (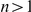, because 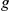 relaxes faster than the expression state).

In summary, a constitutive strategy can confer significantly better growth than responsive expression when the environments are asymmetric in their maladaptation costs or durations. We point out that this is a mere consequence of the finite adaptation times and not of a “cost-of-regulation”.

### Faster growth in random environments

Although periodic environments are common in nature, more generally the environmental durations are random. The passive strategy, having a constant expression level, only experiences the average durations and therefore is not affected by the randomness (environmental durations enter linearly into the time averaged growth rate). For the responsive population, however, it is not clear whether and how randomness will affect its long-term growth.

Here we assume that the individual durations (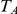,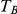) of environments 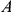 and 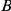 are random and uncorrelated, drawn from exponential distributions with parameters 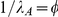 and 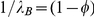.

**Figure 5:**
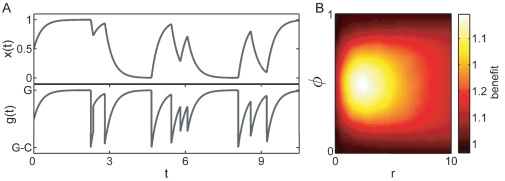
Growth dynamics in random environments. (A) Adaptation dynamics 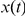 in a random environment (top) and the corresponding momentary growth rate 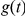 (bottom). During short sporadic environmental changes the phenotype 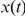 remains close to the previously fit state, and thereby remains adapted for the succeeding environment. As a consequence of the finite adaptation time the population low-pass filters environmental changes and on average spends less time in maladapted states compared to a periodic environment. The time-averaged growth rate in a fluctuating environment significantly exceeds the time-averaged growth rate in a periodic environment. Their ratio defines the benefit in (B). This effect becomes most relevant when the environment on average spends equal amounts of time in both states, and when the response rate 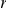 is comparable to the rate of environmental change.

Figure 5A shows the phenotype dynamics (top panel) and the growth rate (bottom panel) for the responsive population as a function of time, according to Eq. 1 to 4 (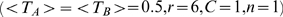). In longer than average conditions the population has time to fully adapt and grow in the optimal phenotype at a maximal rate. During the short environmental conditions, in turn, the phenotype has not enough time to significantly adapt and remains “close” to the previously adapted phenotype. Upon the next environmental change the population can quickly return to the fit state. On average the population thereby spends less time in maladapted states than if every environmental change had a fixed *average* duration. This suggests that the net growth rate should be larger in a random compared to a periodic environment, a condition which had previously been observed in a model of stochastic switching [23].

Since individual environmental durations are random, the population does not periodically cycle along the same phenotype trajectories. To calculate the long-term growth-rate we therefore evaluate it from a large number of cycles of random-durations 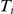. To ensure that the distribution of environmental durations is sampled with sufficient accuracy we choose the number of cycles 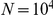. The time averaged growth rate is then obtained by integrating the growth rate over each cycle 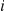 (providing 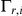 according to Eq. 5) and weighting it in the sum with the fractional duration of the total time. 
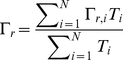
(9)


For a large number of cycles 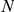 the time-averaged growth rate 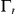 settles at an asymptotic value.

Figure 5B shows the ratio of the long-term growth rate in a random environment (according to Eq. 9) to the long-term growth rate in a periodic environment (obtained according to Eq. 5), where the mean durations in the stochastic and periodic case are identical. For 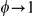 and 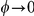 the environment is almost constant, hence there is hardly any fitness difference in this regime. Similarly, if the response rate 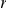 is much larger than the environmental frequency, the difference is small because the population rapidly adapts to even short environmental fluctuations. For 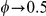 and with response rates comparable to the environmental duration, however, growth in a random environment is significantly faster than in the periodic case, reminiscent of a (stochastically) resonant phenomenon. The net growth rate difference between periodic and random environments depends on the cost of maladaptation 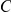: the (on-average) shorter times in maladapted states result in a faster net-growth in the stochastic environment compared to the periodic one. Hence, the greater the cost of maladaptation, the greater is the growth-rate advantage in a random environment (here 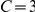). This result illustrates the importance of studying microbial behaviors in a natural setting.

The light gray area together with the white area in Figure 4B indicate the regime in which a response is favored over an optimal constitutive strategy in a random environment. Environmental noise significantly increases the responsive regime. In a random environment the constitutive strategy therefore appears as a good strategy only when environmental changes are sporadic and when responsive regulation is very slow.

### Extrinsic noise benefits the responsive strategy but reduces fitness of the constitutive strategy

Expression of most genes in unicellular organisms is stochastic. As a result, genetically identical cells can show different protein expression levels [36]–[40], adopt different states in the same environment [5], [7], [13], [22], [41], and respond to stimuli with different response times [42], [43]. Different genes show different noise levels, and rather than suppressing noise [44] some cis-regulatory elements seem to promote expression noise [45]–[47]. It therefore is an intriguing question whether and under which conditions inter-cellular variability can provide a benefit or whether noise, as an inevitable side effect of low copy number signaling, always reduces fitness.

We assume that at the beginning of an environmental cycle the population is heterogeneous around the state of its corresponding homogeneous population. Two kinds of population heterogeneity can be distinguished: in the first case individual subpopulations of a responsive population can have different response times 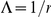 (for mathematical convenience we refer to response times rather than response rates) [42], [43]. In the second scenario different subpopulations are in different states 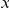. With 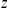 denoting either 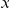 or 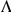, the time-averaged growth rate is 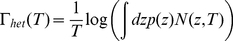
(10)


where the integral is the total population size 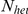 by the time 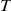 (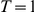 for a full cycle). Here 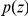 is the distribution of 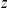, and 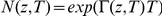 is the change in the size of a subpopulation, cf. Eq. 5 and 6.

**Figure 6:**
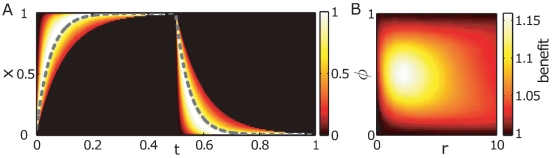
Population dynamics with heterogeneous response rates (A) and benefit compared to a homogeneous population (B). (A) shows the state density as a function of time. During the adaptation phase a population with heterogeneous response rates shows transient heterogeneity in the states 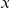. This results in a twofold benefit; i) fast responding subpopulations rapidly adapt and drive the growth of the whole population, whereas ii) for environments 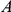 of short duration (small 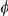) slowly adapting subpopulations remain close to the state that will be fit when 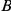 occurs next time. This causes a slight asymmetry of the benefit diagram (B) at large response rates and small 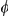 vs. large 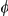. The benefit of heterogeneity, defined as the ratio of heterogeneous and homogeneous population growth rates, is highest when the response rate is comparable to the environmental rate of change.

To ensure that all response times 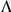 are positive we assume a gamma-distribution for 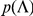. Figure 6A shows the relative frequencies of states in the population as a function of time. The dashed line is for a homogeneous population (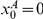, 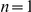, 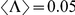, and 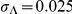). Initially and by the end of the first environment (i.e.,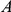) all of the population is in the same state 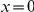, 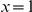 respectively. Due to the heterogeneous response, however, fast responding subpopulations can quickly reach the adapted state and proliferate at a high rate, whereas slowly responding subpopulations are lagging behind the homogeneous one. A heterogeneous response therefore results in transient heterogeneity of the states during the adaptation period. The expected benefit of a heterogeneous response is twofold: first, fast responding subpopulations rapidly adapt and drive population growth at the beginning of the new environment. Second, slowly responding subpopulations remain close to the previously fit state (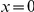 in 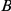) and can quickly resume growth if the environment changes again (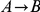) during the adaptation period, i.e. for small 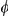.

As a measure of benefit, Figure 6B shows the ratio of the time-averaged growth rates for a heterogeneous and a homogeneous population in environments of different demand 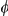, starting with environment 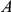. Here 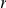 corresponds to the inverse of the average response-time (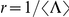). For consistence we keep the coefficient of variation constant for all 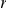 (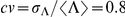). Figure 6B shows that response time variability consistently increases the population fitness, in particular when the average response time is comparable to the cycle duration: clearly, when 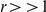 then most cells respond much faster than the environment changes, hence most cells are quasi-instantaneously adapted to a new environment thereby rendering the effect of variability small. On the other hand, if cells respond much slower than the rate of change of the environment, then their state is quasi-constant during a cycle, also decreasing the effect of variability. The slight asymmetry of the benefit at small vs. large 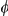, is due to the aforementioned effect of slowly responding subpopulations when the environment rapidly returns to its previous state (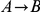 at small 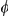). Hence, at short environmental durations slower-than-average responding subpopulations provide a benefit, whereas at long-lasting environmental conditions, the benefit of fast responding subpopulations outweighs the cost of the slowly responding ones.

For a single environmental condition, e.g., 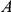 and small variability 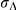, this benefit can be understood straightforwardly. Assuming that the duration of one environmental condition is long enough for all subpopulations to reach the adapted state 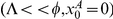 we may write for the population size at time 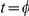


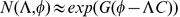
(11)


as follows from Eq. 5. Using Eq. 11 for the integral in Eq. 10 and a normal distribution of response times 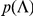 with integration limits from 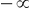 to 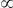 (applicable for small 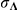), we obtain for the heterogeneous population size at time 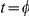


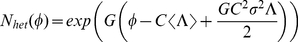
(12)

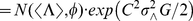
(13)


where we also used the well known gaussian integral. Equation 13 shows that response time variability always provides a benefit after an environmental change compared to a homogeneous population which has the same average response time. This property can be attributed to the convex dependence of the population size 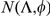 on the response time 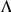 as follows from Jensen's Inequality. In particular we see that the benefit increases with the maladaptation cost 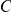,with the steady state growth rate 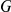,and with the variability 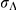. This is a plausible result when considering that the benefit of rapidly adapting cells increases the faster these cells can divide once they have adapted, and that the number of rapidly adapting cells increases with 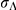.

**Figure 7:**
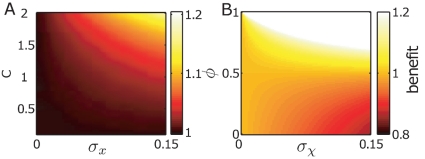
Benefit and cost of state heterogeneity. Benefits and costs are measured by the ratio of heterogeneous and homogeneous population growth rates over one cycle, for a responsive population in (A) and for a constitutive population in (B). For the responsive strategy the benefit of heterogeneity increases with the maladaptation cost and with the variability. The fitness of a constitutive population (B) which is well adapted to environmental cycles where 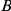 prevails (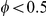) is reduced by variability. Only when the population is significantly maladapted 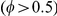 heterogeneity provides a benefit. Note that benefit values in (B) are clipped at 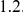

In the second kind of heterogeneity, cells have identical response rates, but noise drives them into different states. When the response is sufficiently fast (and in the absence of multistability) it is reasonable to assume that most cells settle in the optimum state (i.e., 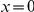 in 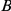) whereas a few cells leak into the nearby states (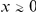). We therefore assume an exponential distribution 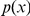 of states at the beginning of a cycle 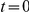 (when the environment switches from 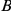 to 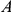). By carrying out the integration in Eq. 10 we obtain the time-averaged growth rate of a heterogeneous population (as our state space is limited to the interval 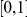, we only consider distributions with a probability 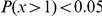). In Figure 7A we compare it to the net growth rate of a homogeneous population where all cells start in 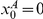, as a function of the maladaptation cost and the variability 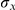 (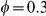). The benefit of state heterogeneity increases with the variability and with the maladaptation cost, clearly because cells that are slightly pre-adapted to the new environment provide a higher benefit when the maladaptation cost is large. On the other hand we found that for 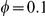 state-variability represents a disadvantage because the benefit of cells that are pre-adapted to condition 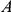 is outweighed by a cost which these cells represent if the environment rapidly changes back to condition 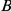 (not shown). Hence expression noise appears to provide a benefit only if different environmental conditions have similar durations, but not when one environment strongly prevails. For a single environmental condition 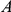 and with 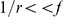 the integral in Eq. 10 can again be carried out analytically, yielding 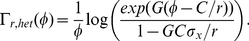
(14)


As we are considering an exponential distribution of states, we have 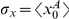. Hence, for a homogeneous population (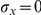) this expression directly explains the increasing benefit at increasing maladaptation costs and variability, after an environmental change. Note that the benefit decreases with increasing response rates, because the population will benefit more from cells that are slightly pre-adapted when the response is slow than when the response is fast. A slowly responding population might therefore increase its fitness by increasing gene expression noise. We mention that the results remain qualitatively similar when we assume a symmetric distribution around an initial state 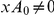.

Figure 7B shows the ratio of the time-averaged growth rates of a constitutive-heterogeneous to a constitutive-homogeneous population as obtained from Eq. 10. We assumed that the constitutive population is optimized for growth in environmental cycles where 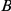 prevails 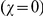, and has an exponential distribution 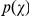 over neighboring states 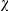. For the case that the homogeneous population is reasonably well adapted (i.e. for 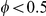), heterogeneity represents a significant cost because a smaller fraction of the population resides in the optimum state (this is similar to a responsive population in an environment where one condition strongly prevails). Only if the population is sufficiently maladapted (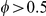), diversification can increase fitness due to the presence of a small well-adapted subpopulation.

Gene expression levels can evolve to an optimum within a few hundred generations [16]. The above results therefore indicate that the expression of a constitutive gene will be selected against noise [39], [48]–[50]. On the other hand, we find that a responsive strategy can benefit both, from heterogeneous states and from heterogeneous response times, in particular when maladaptation costs are high and when both environmental durations are comparable to the population-averaged response time.

## Discussion

It is a general belief that responding to an environmental change is better than not responding. It is not a priori clear, however, whether responding is indeed the best strategy in a rapidly changing environment. In fact, many genes are not responsively regulated but expressed constitutively despite a varying demand for the gene product. In this article we explained which conditions select for a constitutive or a responsive gene expression strategy in a time-varying environment, taking account of environmental and inter-cellular noise.

With a responsive strategy a population can switch between two adapted phenotypes, where each one confers maximal growth in one environment while minimizing growth in the other, cf. Figure 1. After an environmental change the responsive population is maladapted and requires time for the transition into the adapted state, eventually reaching it by a time when the environment changes once again. With a constitutive-passive strategy a population can evolutionarily tune its phenotype to an optimal intermediate level, which on one hand allows suboptimal (intermediate) growth at all times in both environments [16], and which on the other hand bypasses the adaptation lag. As a function of the maladaptation cost, the time scales of environmental changes, and of the genetic response we have studied which is the optimal constitutive expression-level and under which conditions it confers faster growth than a responsively regulated expression.

We found that the optimal constitutive level in a changing environment is different from the average optimal expression level (Figure 3): when the growth rate is a convex function of the expression level, the optimum maximizes growth in one environment while providing minimal growth in the other. When the growth rate is a concave function, the optimal constitutive level is an intermediate one, providing intermediate growth in both environments. Interestingly, whether convex or concave, the optimum level is generally different from the average demand for the gene product.

At large maladaptation costs the constitutive strategy confers net-growth only when one of the environmental conditions prevails, otherwise a fast responding strategy becomes imperative to achieve net growth. A responsive population that cannot respond sufficiently fast, however, lags behind the environment: its expression state slowly oscillates around the averaged demand for the gene product and does not reach the optimum in any of the two environments (cf. Figure 1C). Under these conditions constitutive optimal expression provides a larger time-averaged growth rate than responsively regulated expression, cf. Figure 4.

The responsive vs. constitutive regimes are separated by a first order phase-transition. This indicates that a fast genetic response cannot evolve starting from constitutive expression via a slow response, because it would have to go through a regime of lower fitness. This condition may give rise to evolutionary hysteresis as recently suggested for the evolution of stochastic switching [24]. An interesting question that arises, is how responsive gene expression can evolve from constitutive expression.

Previous studies [6], [33] had found that constitutive expression can only be better than responsive expression if the cost for sensing and regulatory machinery is high. In other words, when regulation comes “for free” these studies predict that regulation will always be selected for. In striking contrast, we neglected the cost of regulation and still find that constitutive expression can be better than adaptive expression. This result is a mere consequence of explicitly taking into account the (slow) adaptation dynamics and the intermediate states, which were neglected in previous studies.

We find that a responsive strategy has significantly larger growth rates in random environments compared to periodic environments when the time scales of the genetic response and environmental change are comparable, see Figure 5B. A similar effect was previously observed in a model of stochastic switching [23], therefore it would be very interesting to verify this prediction experimentally. Furthermore we find that in a changing environment a responsive strategy can benefit from inter-cellular noise, in particular when environmental durations are comparable to the population averaged response time, whereas the fitness of the constitutive strategy is impaired, cf. Figure 6 and 7.

Thus, our main conclusions are: *i)* that a constitutive gene-expression strategy is better than a responsive strategy when the environments are asymmetric or when a response is not sufficiently fast and *ii)* that constitutively expressed states are selected against noise whereas genes that respond to environmental changes may benefit from noise.

Recent analysis of yeast high-throughput data indeed confirm this result [39], [48]–[50]: genes which are constitutively expressed and under an almost constant demand (commonly referred to as housekeeping genes) have below-average levels of gene expression noise, the proteasome having the least [39]. This is in agreement with other theoretical works on gene expression in a constant environment [17], [35], [35,51]. Tightly regulated genes, which respond to environmental perturbations, however, show systematically higher levels of gene expression noise. This is particularly striking for stress resistance genes and for the products of metabolic systems in the repressed state [39], [42], [46], [49]–[52]. In principle the noise levels can be tuned by the cell [36]; therefore it would be interesting to experimentally verify a correlation between gene expression noise, gene response time, and the frequency of demand for a gene product. More specifically this may be achieved in a laboratory evolution experiment where a noisy gene that responds to environmental perturbations is put under constant demand. According to our analysis evolution will then select against gene expression noise.

Complementing previous works on phenotypic switching [6], [23]–[25] our results allow to divide the environmental parameter space into three regimes of optimal growth strategies: a) When populations can rapidly switch between adapted states the responsive switching strategy is the best. b) When adaptation is slow and environments are symmetric, stochastic switching is preferred over responsive switching [23]–[25]. c) When a response is slow and the environment is asymmetric, constitutive expression is better than responsive switching (this study), whereas stochastic switching can be worse [24], [25].

Our predictions on optimality of constitutive expression can be verified experimentally as follows. In a constant lactose environment with saturating inducer concentrations, the expression level of the *lac-*operon was shown to adapt to an optimum within a few hundred generations [16]. Using a similar protocol the constitutive mutants, 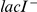 or 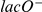, can be evolved in a changing environment where the demand for *Lac* proteins oscillates in time. Our method predicts the (optimal) expression levels to which a constitutively expressing strain will evolve as a function of the expression demand 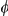.

Recently developed promoters allow for a graded induction of various sugar systems [53], [54]. By varying the inducer concentration these promoters can be used to measure the growth-rate dependence on the expression level of different genes (i.e., the cost-benefit relationship) and to determine the optimal constitutive expression levels at different demands for the gene product 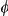. It would also be very interesting to use these promoters to characterize a large set of cost-benefit functions: do all functions fall into a certain class? Are there threshold-like cost-benefit functions? Classifying and understanding the shape of these functions may provide profound insights into cellular expression regulation.

Using microfluidic devices [55] the time-averaged growth rates in a rapidly changing environment can be measured [22, 34, allowing comparison to our constitutive vs. responsive strategy diagrams, see Figure 4B. For *E. coli*, using the experimentally determined cost-benefit data of the *lac-*operon (not expressing LacZ when lactose is available: 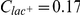, expressing LacZ when lactose is unavailable 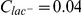, 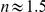, at 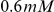 [16]) our analysis predicts that constitutive *lac-*expression will have a growth rate advantage when the expression demand lies above 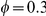 at an environmental cycle duration 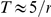 (where 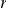 is the response rate of the lac-operon). When the cycle duration is longer, e.g. 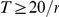, then the responsive strategy has sufficient time to fully adapt and the intermediate-constitutive strategy will only have higher fitness when the demand for lac-expression also increases above 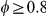. Together this indicates that the lac-operon was optimized for rare lactose availability with long cycle durations, consistent with previous works [16], [17]. Indeed, in a natural setting *E. coli* finds lactose only during 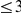 hours while traversing the primary mammalian intestine [1], whereas lactose is unavailable in most other habitats (colon, soil, water). Assuming a cycle duration 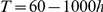 (hence the demand 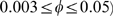), our analysis consistently predicts that regulated-induction of *Lac*-proteins confers higher fitness than optimal constitutive expression.

Finally, a comparison of regulatory strategies across different species that evolved in different habitats would provide further insight into the interplay of environmental demand frequency and the requirements for the regulation of genes [1]. Specifically, constitutive gene expression levels and gene induction patterns may differ significantly between the wild type *S. Cerevisiae* populations, and populations which were used over many generations in industrial fermenters, e.g., breweries.

In this paper we have studied optimal gene expression strategies in a rapidly changing environment. We analyzed the interplay between the timescales of genetic response and the demand for a phenotype, the maladaptation costs, and the fitness. Some of our predictions agree with experimental observations, and we suggest the experiments needed to verify others. We believe this will stimulate further experimental work and – in line with our predictions – deepen our understanding of microbial gene expression strategies.
